# Determinants of mortality status and population attributable risk fractions of the North West Province, South African site of the international PURE study

**DOI:** 10.1186/s13690-024-01336-y

**Published:** 2024-07-05

**Authors:** Cristian Ricci, Iolanthe M. Kruger, Herculina S. Kruger, Yolandi Breet, Sarah J. Moss, Abie van Oort, Petra Bester, Marlien Pieters

**Affiliations:** 1 Africa Unit for Transdisciplinary Health Research, Faculty of Health Sciences, North- West University, Potchefstroom, South Africa; 2 Centre of Excellence for Nutrition, Faculty of Health Sciences, North-West University, Potchefstroom, South Africa; 3 SAMRC Extramural Unit for Hypertension and Cardiovascular Disease, Faculty of Health Sciences, North-West University, Potchefstroom, South Africa; 4 Centre of Excellence for Hypertension in Africa Research Team, Faculty of Health Sciences, North-West University, Potchefstroom, South Africa; 5 Physical activity, Sport and Recreation Research Focus Area, Faculty of Health Sciences, North-West University, Potchefstroom, South Africa

**Keywords:** Cause of death, Mortality, Comparative risk assessment, Population-attributable fractions, African

## Abstract

**Background:**

Mortality data and comparative risk assessments from sub-Saharan Africa are limited. There is an urgent need for high quality population health surveys to be conducted, to improve the national health surveillance system. Our aim was to perform a comparative risk assesment and report on the mortality status and cause of death data of participants from a South African site of the international Prospective Urban Rural Epidemiology study.

**Methods:**

1 921 Black participants were included, with a median observational time of 13 years resulting in 21 525 person-years. We performed a comparative risk assessment considering four health status domains: locality (rural vs. urban), socio-economic status (SES) (education and employment), lifestyle factors (physical activity, smoking and alcohol consumption) and prevalent diseases (human immunodeficiency virus (HIV), type 2 diabetes mellitus and hypertension). Next, population-attributable fractions (PAFs) were calculated to determine the mortality risk attributable to modifiable determinants.

**Results:**

577 all-cause deaths occurred. Infectious diseases (28.1% of all deaths) were the most frequent cause of death, followed by cardiovascular disease (CVD) (22.4%), respiratory diseases (11.6%) and cancer (11.1%). The three main contributors to all-cause mortality were HIV infection, high SES and being underweight. HIV infection and underweight were the main contributors to infectious disease mortality and hypertension, the urban environment, and physical inactivity to CVD mortality. HIV had the highest PAF, followed by physical inactivity, alcohol and tobacco use and hypertension (for CVD mortality).

**Conclusion:**

This African population suffers from a quadruple burden of disease. Urban locality, high SES, prevalent disease (HIV and hypertension) and lifestyle factors (physical inactivity, tobacco and alcohol use) all contributed in varying degrees to all-cause and cause-specific mortalities. Our data confirm the public health importance of addressing HIV and hypertension, but also highlights the importance of physical inactivity, tobacco use and alcohol consumption as focal points for public health strategies to produce the most efficient mortality reduction outcomes.

**Supplementary Information:**

The online version contains supplementary material available at 10.1186/s13690-024-01336-y.

**Table:** 

Text box 1. Contributions to the literature	
• Mortality data and comparative risk assessments from sub-Saharan Africa are limited and high quality population health surveys are therefore, needed. • This study provides much-needed local empirical data necessary for the prioritisation and identification of appropriate health promotion, interventions and disease prevention programmes in South Africa to produce the most profitable mortality reduction outcomes. • While our data confirm the public health importance of addressing HIV and hypertension, it also highlights the importance of physical inactivity, tobacco use and alcohol consumption as focal points in public health promotion programmes and disease prevention strategies.	

## Introduction

The international Prospective Urban Rural Epidemiology (PURE) study was designed to determine the impact of societal influences on non-communicable disease (NCD) risk factors, morbitidy, and mortality in low-, middle- and high-income countries [1]. The motivation for the PURE study stems largely from the difference in health status observed between high-income (early industrialisers) and low- and middle-income (late industrialisers) countries, particularly with regard to NCDs. In high-income countries, there was a decline in deaths from infectious and childhood diseases and an increase in NCDs during the 20th century [2]. This was the result of economic development, industrialisation, and urbanisation, which improved living conditions, medical treatment, sanitation, and economic growth [3]. In low- and middle-income countries, however, urbanisation took place much later and largely occurred without the accompanying economic growth and infrastructure development, resulting in urban slums and more socio-economic inequality [4]. Moreover, the urban transition is accompanied by changes in socio-economic status (SES), lifestyle and prevalent diseases (degenerative vs. infectious) that differ distinctly from the rural environment [3, 5].

South Africa is a middle-income country, and one of four African countries included in the international PURE study. Like most other low- and middle-income countries, South Africa not only suffers from a high prevalence of NCDs, but a large proportion of the population suffers from a quadruple burden of disease. These are (1) infectious diseases such as the human immunodeficiency virus (HIV) / acquired immunodeficiency syndrome (AIDS) and tuberculosis; (2) high maternal and child mortality; (3) high levels of violence and injuries; as well as (4) NCDs [6], with some changes in disease burden trajectories, reported over recent years [7, 8]. However, mortality data and comparative risk assessment, which provides estimates of the relative contribution of risk factors, from sub-Saharan African countries are limited. Findings from the Second Comparative Risk Assessment Study of South Africa [8] highlight the need for high quality population health surveys to be conducted, to improve the national health surveillance system.

The aim of this study is to report on the mortality status and cause-of-death data at 13 years of follow-up of participants from the North West Province PURE-study site, one of two sites in South Africa. First, we perform a comparative risk assessment considering four main domains of health status: locality (rural vs. urban environment), SES (education and employment), lifestyle factors (physical activity, smoking and alcohol consumption) and prevalent diseases (HIV, type 2 diabetes mellitus (T2DM) and hypertension). Lastly, population-attributable fractions are calculated to determine the population-level mortality risk attributable to modifiable determinants.

## Methods

### Study design and sample collection

**Fig. 1:**
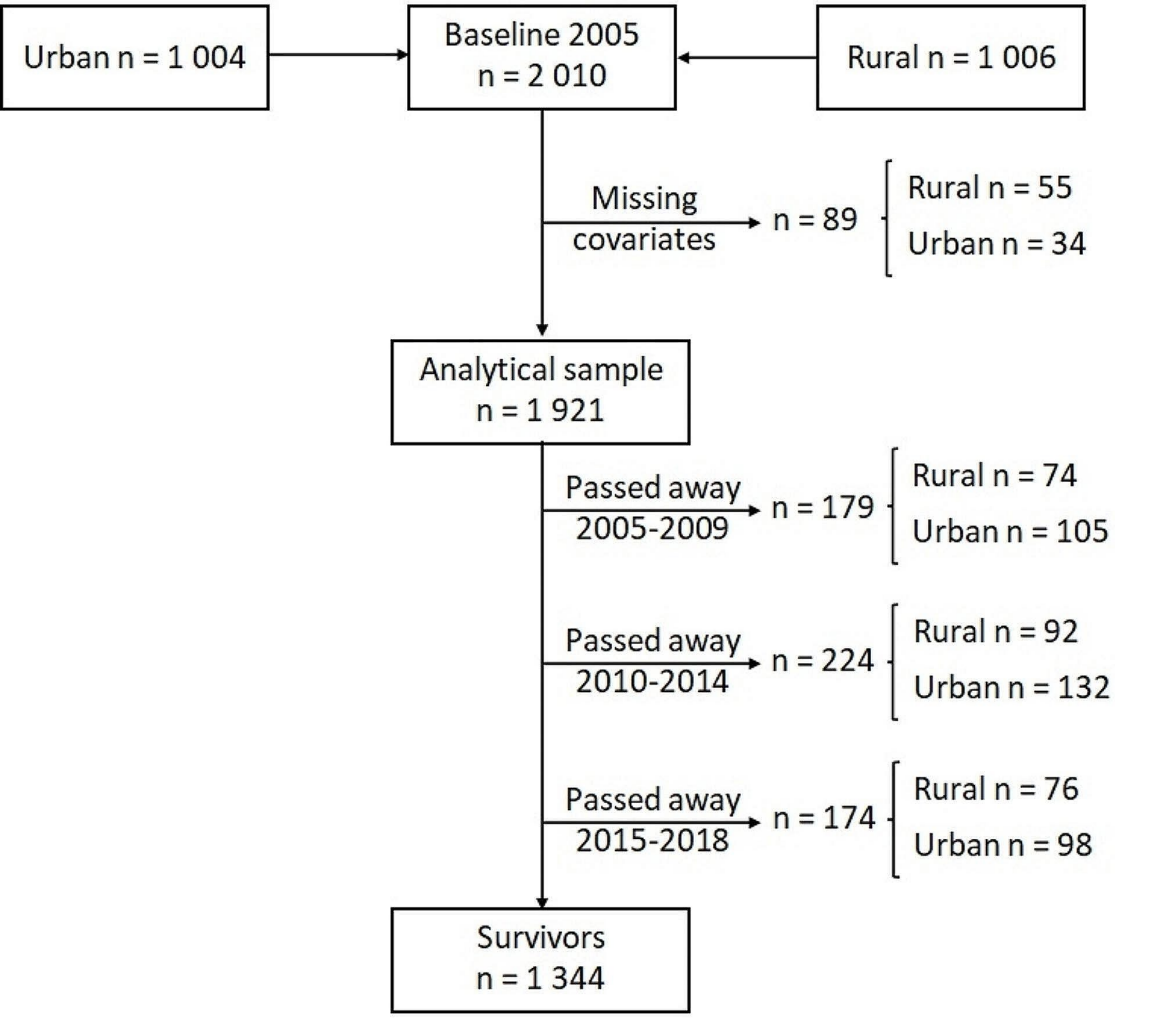
Participant flow chart

The PURE study is an international, prospective, epidemiological study that determines changes in lifestyle, risk factors and incidence of chronic diseases in 27 low-, middle-, and high-income countries [1]. This study reports on data collected from the North West Province PURE study site in South Africa. Black men and women older than 30 years were eligible for inclusion. Any self-reported prior cardiovascular event, acute illness, pregnancy, or lactation was considered basis for exclusion. A stratified random sample from *N* = 6 000 randomly selected households in distinct rural and urban communities was included. The urban stratum was defined as people living in established townships and informal settlements in close proximity to a major city, while the rural stratum was defined as people living under tribal law ≥ 50 km from urban centres. Migration stability within the chosen community over the study period was an additional requirement. The study was approved by the Health Research Ethics Committee of the North-West University, South Africa (04M10 and NWU-00016-10-A1) and complies with the revised Helsinki Declaration. Written informed consent was obtained from all participants. Baseline data were collected in 2005 (*N* = 2 010; 1 006 rural and 1 004 urban participants). Mortality data is available up to 2018 (13-year follow-up) (Fig. 1).

### Socio-economic status, lifestyle, and prevalent diseases

Data regarding SES, self-reported alcohol, tobacco, and medication use were collected using a standardised questionnaire. Physical activity was determined using an adapted BAECKE questionnaire validated for the Black population of the North West Province [9], where participants reported physical activity for commuting, work, leisure and sport participation. Body-mass-index (BMI) was calculated from weight (measured to the nearest 0·01 kg) divided by height (as meters, measured to the nearest 0·1 cm) squared (kg/m^2^). Voluntary human HIV testing was performed with a first response rapid HIV card (PMC Medical, India) and, if positive, confirmed with a further test (Pareeshak card test, BHAT Bio-tech, India). Registered HIV counsellors provided pre and post–test counselling. Brachial blood pressures were measured in the supine position after 5 min of rest, with the second of two measures recorded, using the Omron HEM-757 device (Omron Healthcare, Kyoto, Japan). Hypertension was defined according to the 2018 European Society of Cardiology / European Society of Hypertension guidelines [10] as a systolic and/or diastolic blood pressure equal to or greater than 140/90 mmHg, or the use of antihypertensive medication. Fasting plasma glucose from samples collected in fluoride tubes was measured with a hexokinase method using the Synchron^®^ System(s) (Beckman Coulter Co., Fullerton, CA, USA) and reagents. Haemoglobin A1c (HbA1c) was measured using the D-10 Haemoglobin Testing System (Bio-Rad, California, USA). For this paper, fasting blood glucose ≥ 7 mmol/L and/or HbA1c ≥ 6.5% or the use of hypoglycaemic agents were used to indicate the presence of T2DM [11, 12].

### Coding of exposures and outcomes

Four main domains of health status, coded by nine key exposure variables, were considered to estimate mortality risk. Firstly, we investigated locality and socio-economic status since these exposures may represent independent proxies of access to health resources and social inequality. Locality was coded as rural or urban, according to the design of the international PURE study [1]. SES was coded according to self-reported education and being employed (high SES was coded as the presence of both education above grade 9 and being employed). Lifestyle was the third key exposure that was considered. Specifically, we coded lifestyle by physical activity (physically active was coded as highest quartile of the physical activity index), self-reported ever use (current and previous users combined) of alcohol and tobacco and by BMI categories (coded as underweight (BMI < 18.5 kg/m^2^), normal weight (18.5 kg/m^2^ ≤ BMI < 25 kg/m^2^), overweight (25 kg/m^2^ ≤ BMI < 30 kg/m^2^) and obese (BMI ≥ 30 kg/m^2^)). Finally, the presence of prevalent diseases, namely HIV, hypertension and T2DM, was considered as the fourth domain of overall health status. For this paper, fasting blood glucose ≥ 7 mmol/L and/or HbA1c ≥ 6.5% or the use of hypoglycaemic agents were used to indicate the presence of T2DM [11, 12]. The above-mentioned lifestyle factors and prevalent diseases were chosen as they are widely acknowledged as the leading health threats in South Africa [13].

Mortality data, as recorded on the participants’ death certificates provided by Statistics South Africa, was the outcome considered in the present work. The first analysis considered all-cause mortality. Next, the four most prevalent main classes of underlying cause of mortality, according to the International Classification of Diseases’ 10th revision (ICD10) coding, were identified. These were: infectious diseases (ICD10 = A00-B99), CVD (ICD10 = I00-I99), respiratory diseases (ICD10 = J00-J99) and cancer (ICD10 = C00-D48).

### Statistical methods

Continuous data were described using the median and the 5th to 95th percentiles, while categorical data were reported as counts and percentages. Univariate tests were conducted to compare sex. All-cause and cause-specific mortality risks were estimated using a time-to-event analysis based on a Cox model with age-to-event as the underlying time metric and having 10-year age classes and sex as strata variables. Factors under investigation were locality (urban or rural area), SES, physical activity, ever use of tobacco or alcohol, HIV, hypertension, and T2DM. Hazard ratios (HR) and 95% CI were used to depict all-cause and cause-specific mortality risks in both simple models considering the aforementioned factors individually and by mutually adjusted models considering all factors combined so that each HR was computed by average values of all the other determinants. Finally, we performed an estimate of population attributable fraction percentage (PAF%) according to the cohort design. Briefly, we used the relative risk estimates of all-cause mortality and cause-specific mortality in combination with the determinants’ prevalence in the sample, performing the mortality reduction due to the elimination of a given exposure while the distribution of other modifiable and non-modifiable risk factors is unchanged [14].

Supplementary analyses were conducted stratified by sex. A sensitivity analysis was also conducted, excluding participants who died in the first year of observation. The proportionality of hazards was investigated by visual inspection of Schoenfeld’s residuals. All statistical tests were two-tailed and were performed based on a type-I error rate of 5% (α = 0.05). SAS vers.9.4 was used for all statistical analyses. The calculation of the PAF% was conducted using the %par macro programme.

### Determination of the sample size adequacy

The numDEpi.default function of the powerSurvEpi library of the R software was used to compute the required sample size. According to our calculation, the present sample size and number of events (irrespective of considering cause-specific or all-cause mortality) were sufficient to detect a hazard ratio above 1.5 with a statistical power above 80% (1-β ≥ 0.8) considering a type-I error of 5% (α = 0.05). For stratified analysis based on sex, the resulting sample size and number of events (irrespective of considering cause-specific or all-cause mortality) were sufficient to detect a hazard ratio above 2.2 with a statistical power above 80% (1-β ≥ 0.8) considering a type-I error of 5% (α = 0.05).

## Results

In the present study, 1 921 participants from the PURE study had sufficient information to be included in the analytical data frame. The median age at recruitment was 48 years (36 to 68 years), and most participants were women (62.6%). The analytical sample was composed of a similar proportion of participants from rural (*N* = 951; 49.5%) and urban (*N* = 969; 50.5%) areas. Regarding employment and education, 1 314 (68.4%) participants were employed and received education higher than grade 9. The median physical activity index of the analytical sample was 7.3 (4.6–10.1). This equates to middle level physical activity (e.g. factory work, carpentry, farming, hospital nurse, plumber or house cleaning), and the median BMI was 23.0 kg/m^2^ (16.3–38.4 kg/m^2^). Most notably, many participants were underweight (*N* = 847; 44.1%). A similar proportion of participants were normal weight (*N* = 318; 16.5%), overweight (*N* = 339; 17.7%) or obese (*N* = 416; 21.7%). However, when looking at the BMI distribution by sex, women had a median BMI of 26.0 kg/m^2^ (16.8–40.8 kg/m^2^), and men a median BMI of 19.8 kg/m^2^ (15.9–28.1 kg/m^2^) (*P* < 0.0001). Regarding other behavioural factors, there was a high rate of unhealthy behaviours, with most participants ever-using tobacco (*N* = 1 260; 65.8%) or alcohol (*N* = 983; 51.4%). Finally, almost half of the participants had hypertension (*N* = 907; 47.6%) and 399 (20.8%) participants were HIV infected or had T2DM (*N* = 102; 5.3%). Overall, the participants were followed up for a median observational time of 13 years resulting in 21 525 person-years. Descriptive data according to mortality status is reported in Table 1. During this observation period, 577 (30%) all-cause deaths occurred. Regarding specific causes of mortality, infectious diseases (*N* = 162; 8.4% of participants; 28.1% of all deaths) were the most frequent cause of mortality, followed by CVD (*N* = 129; 6.7% of participants; 22.4% of all deaths), respiratory diseases (*N* = 67, 3.5% of participants; 11.6% of all deaths) and cancer (*N* = 64, 3.3% of participants; 11.1% of all deaths). When looking at the age of the different mortality causes, the median age at event for all-cause mortality was 59.3 years (41.2–82.3 years), 53.2 years (39.6–72.6 years) for infectious diseases, 64.9 years (48.3–84.7 years) for CVD, 60.2 years (40.5–76.6 years) for respiratory diseases and 59.8 years (46.9–76.3 years) for cancer mortality.

**Table 1:** Characteristics of the study participants

	Survivors (*N* = 1,344)	All deaths (*N* = 577)	Infection deaths (*N* = 162)	CVD deaths (*N* = 129)	Respiratory deaths (*N* = 67)	Cancer deaths (*N* = 64)
Age (years)	47.0 (36.0; 67.0)	52.0 (37.0; 74.0)	46.0 (36.0; 66.0)	57.0 (43.0; 75.0)	54.0 (36.0; 72.0)	53.5 (38.0; 72.0)
Sex (women)	908 (67.6)	294 (51.0)	83 (51.2)	71 (55.0)	30 (44.8)	30 (46.9)
Follow-up (years)	13.2 (12.7; 13.6)	6.6 (0.7; 12.4)	6.6 (0.7; 12.3)	7.2 (1.3; 12.4)	5.7 (0.5; 10.9)	7.1 (1.2; 12.7)
Age at event (years)	59.9 (49.0; 79.8)	59.3 (41.2; 82.3)	53.2 (39.5; 72.6)	64.9 (48.3; 84.7)	60.2 (40.5; 76.6)	59.8 (46.9; 76.3)
Locality (rural)	709 (52.8)	242 (41.9)	72 (44.4)	44 (34.1)	25 (37.3)	26 (40.6)
High SES	899 (66.9)	416 (72.1)	122 (75.3)	88 (68.2)	41 (61.2)	46 (71.9)
PAI	7.6 (4.7; 10.2)	6.4 (4.3; 9.7)	6.3 (4.5; 9.7)	6.1 (4.1; 9.5)	6.1 (4.6; 9.6)	6.8 (4.0; 9.7)
Physically inactive	275 (20.5)	206 (35.7)	54 (33.3)	55 (42.6)	29 (43.3)	23 (35.9)
Ever smoker	849 (63.3)	412 (71.5)	120 (74.1)	86 (66.7)	55 (82.1)	47 (73.4)
BMI (Kg/m^2^)	23.8 (16.9; 39.1)	21.2 (15.6; 36.3)	19.8 (15.8; 33.8)	23.2 (16.1; 36.4)	19.0 (15.1; 30.6)	21.3 (15.7; 33.1)
Underweight	529 (39.4)	318 (55.1)	110 (67.9)	52 (40.3)	48 (71.6)	34 (53.1)
Normal weight	227 (16.9)	91 (15.8)	20 (12.4)	29 (22.5)	8 (11.9)	10 (15.6)
Overweight	254 (18.9)	86 (14.9)	16 (9.9)	24 (18.6)	7 (10.5)	13 (20.3)
Obese	334 (24.9)	82 (14.2)	16 (9.9)	24 (18.6)	4 (6.0)	7 (10.9)
HIV	241 (18.0)	159 (27.7)	84 (52.2)	13 (10.1)	13 (19.4)	13 (20.3)
Hypertension	589 (44.2)	319 (55.5)	82 (50.6)	94 (73.4)	32 (47.8)	28 (43.8)
Type-II diabetes	63 (4.7)	39 (6.8)	5 (3.1)	11 (8.5)	2 (3.0)	3 (4.7)

### Determinants of all-cause and cause-specific mortality

**Fig. 2:**
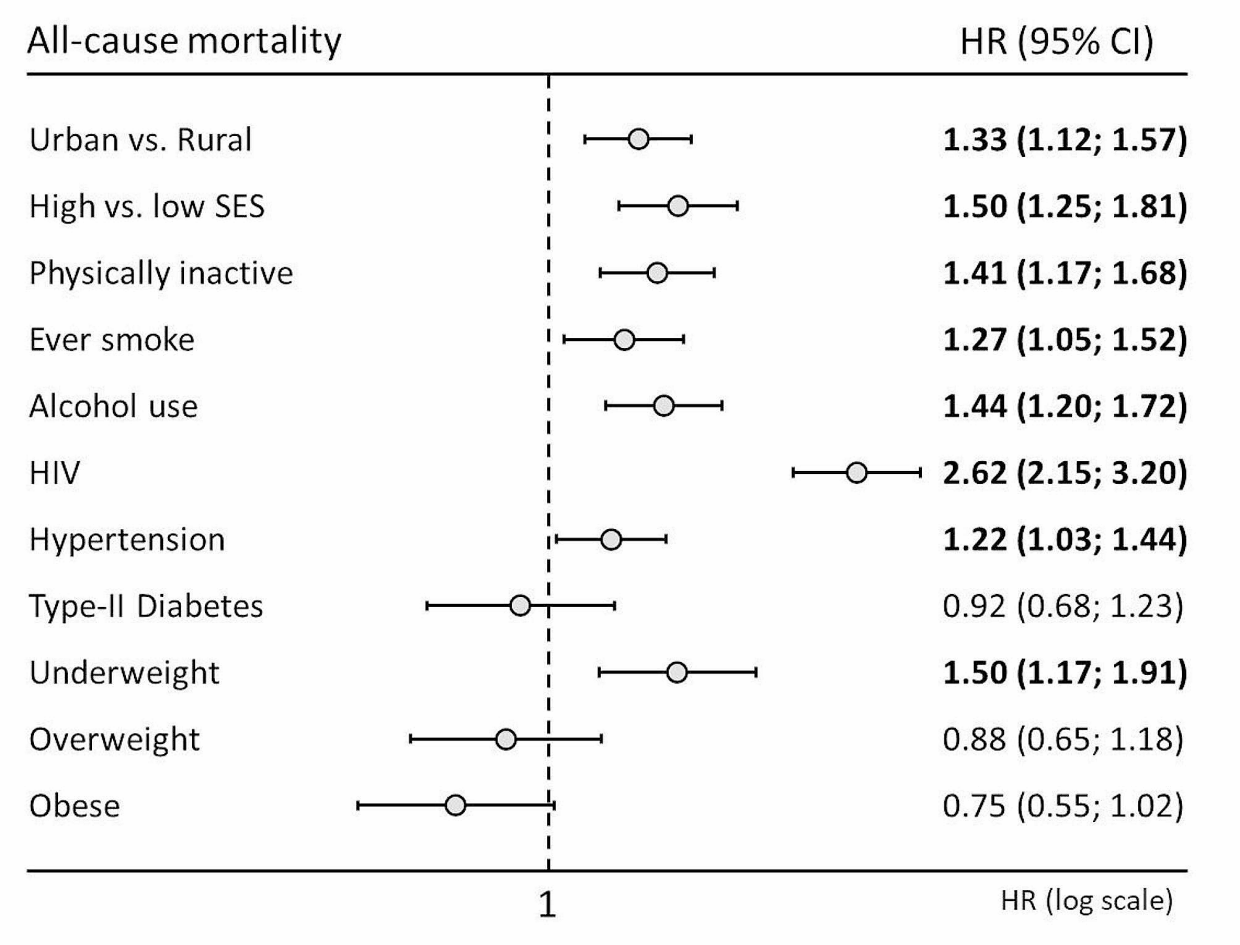
Hazard ratio and 95% confidence interval for all-cause mortality. The HR for BMI categories was performed considering normal weight (BMI = 18.5–25 kg/m^2^) as reference category

When looking at the analysis performed for all-cause mortality, HIV infection (HR = 2.62; 2.15–3.20), high SES (HR = 1.50; 1.25–1.81), being underweight (HR = 1.50; 1.17–1.91), ever use of alcohol (HR = 1.44; 1.20–1.72), being physically inactive (HR = 1.41; 1.17–1.68), living in an urban area (HR = 1.33; 1.12–1.57), ever use of tobacco (HR = 1.27; 1.05–1.52), and hypertension (HR = 1.22; 1.03–1.44) were related to between a 50% to two and a half fold increased risk of mortality (Fig. 2).

**Fig. 3:**
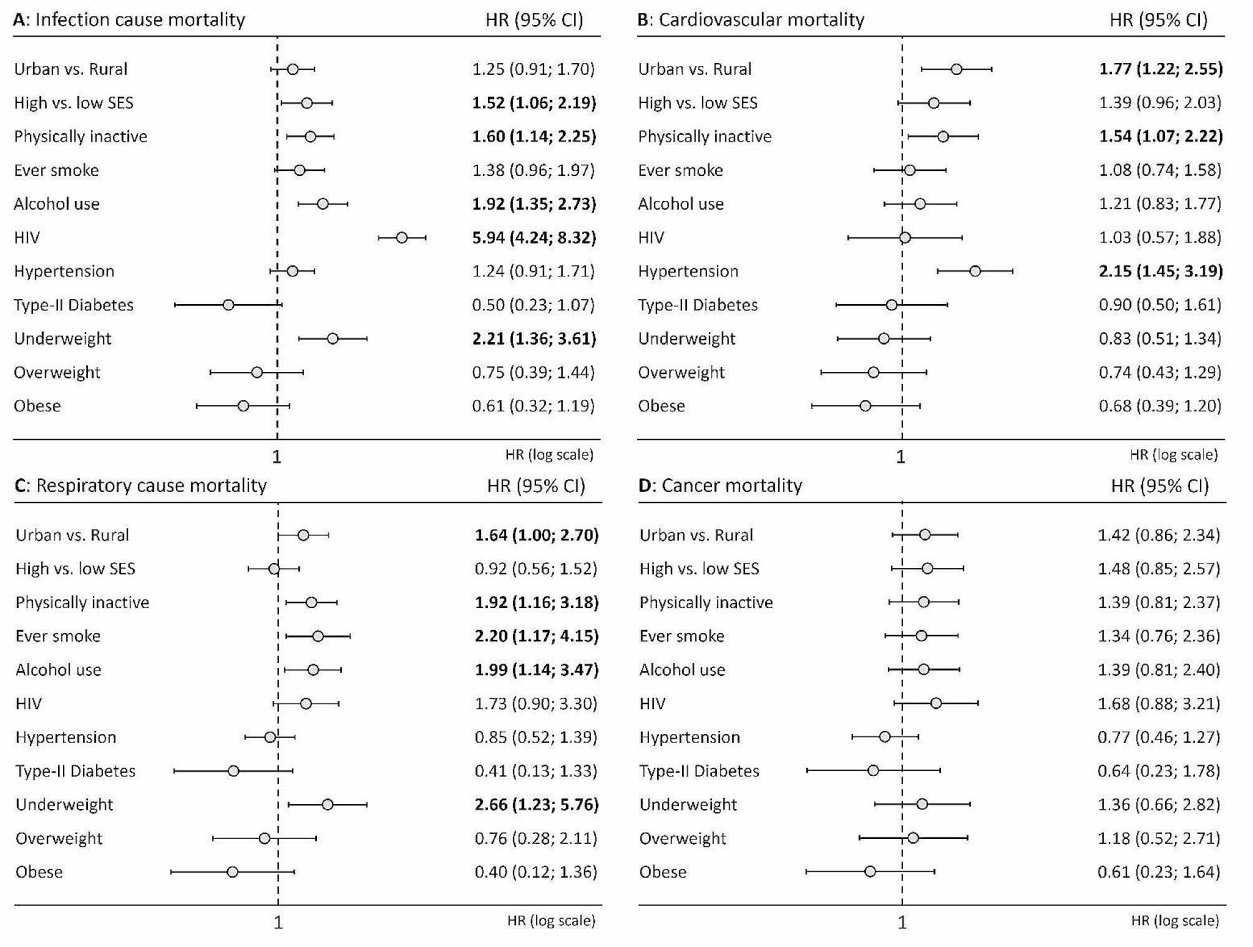
Hazard ratio and and 95% confidence interval for cause-specific mortality. The HR for BMI categories was performed considering normal weight (BMI = 18.5–25 kg/m^2^) as reference category

Distinct determinants of death emerged when looking at cause-specific mortality. For infectious disease mortality (Fig. 3A), we observed a 50% to almost six-fold increased mortality risk for HIV infection (HR = 5.94; 4.24–8.32), being underweight (HR = 2.21; 1.36 to 3.61), ever alcohol use (HR = 1.92; 1.36–2.73), being physically inactive (HR = 1.60; 1.14–2.25), and high SES (HR = 1.52; 1.06–2.19). Regarding CVD mortality (Fig. 3B), there was a more than two-fold increased mortality risk for being hypertensive (HR = 2.15; 1.45–3.19). We also found increased mortality risk for participants living in urban areas (HR = 1.77; 1.22–2.55) and for being physically inactive (HR = 1.54; 1.07–2.22). In terms of respiratory disease mortality (Fig. 3C), there was an increased mortality risk of about two to two and a half fold for being underweight (HR = 2.66; 1.23–5.76), ever tobacco use (HR = 2.20; 1.17–4.15), ever alcohol use (HR = 1.99; 1.14–3.47), and being physically inactive (HR = 1.92; 1.16–3.18). Finally, no statistically significant association was observed for any of the factors considered in relation to cancer mortality (Fig. 3D).

### Analyses of mutually adjusted models

For all-cause mortality, the above-reported significant associations with HIV infection (HR = 2.52; 2.06–3.08), high SES (HR = 1.55; 1.28–1.87), being underweight (HR = 1.50; 1.17–1.92), being physically inactive (HR = 1.27; 1.03–1.56), and having hypertension (HR = 1.21; 1.02–1.45) were confirmed. On the contrary, the significant associations reported above for living in a rural area, and ever tobacco and alcohol use were lost. When considering the mutually adjusted model of mortality due to infectious diseases, an increased mortality risk for being HIV infected (HR = 5.33; 3.78–7.51), underweight participants (HR = 2.19; 1.33–3.61), for higher SES (HR = 1.70; 1.17–2.46), and for being physically inactive (HR = 1.51; 1.01–2.24) were confirmed. For CVD mortality, the mutually adjusted model confirmed the increased CVD mortality risk of being hypertensive (HR = 2.07; 1.38–3.10) but not the reduced risk for physically active participants. Regarding respiratory diseases, only an increased mortality risk for underweight participants (HR = 2.45; 1.12–5.37) was confirmed in the mutually adjusted model. Finally, in terms of cancer mortality, the absence of any statistically significant associations was confirmed in the mutually adjusted model.

### Analysis by sex and sensitivity analysis

The above-reported results were largely confirmed by analyses conducted by sex and after exclusion of participants who died in the first year of observational time (Supplementary Tables 1 and 2). The results confirmed the protective role of physical activity, and the detrimental role of alcohol intake and HIV infection for all-cause mortality in both men and women. When looking at specific causes of mortality, HIV infection was confirmed as the leading determinant of mortality due to infectious diseases in both men and women. For CVD mortality, physical activity and rural locality were protective in women but not in men, while hypertension was confirmed as the most relevant determinant in men. When looking at respiratory disease mortality, being underweight was the leading determinant in men but not in women, for whom (ever) alcohol use was related to an almost 3-fold increased mortality risk.

### Public health impact of modifiable mortality determinants

Regarding modifiable behavioural determinants in relation to all-cause mortality, we observed that eradicating HIV would reduce mortality by 23.2% (17.4–28.8%) (Table 2). Cessation of tobacco use and alcohol consumption would result in a consistent all-cause mortality reduction of 14.9% (3.6–25.8%) and 18.1% (9.1–26.8%), respectively. Furthermore, increasing physical activity and eradicating hypertension would result in an all-cause mortality reduction of 9.7% (4.2–15.1%) and 9.6% (1.3–17.9%), respectively. Finally, eliminating underweight would result in a 17.5% (6.7–27.9%) reduction in all-cause mortality. The above results were partially confirmed when looking at specific causes of mortality. For mortality due to infectious diseases, we estimated that HIV alone contributed to almost half (47.9%; 37.2–57.3%) of the mortality. Acting against physical inactivity, alcohol consumption, and underweight would reduce infectious disease mortality by 13.7% (3.0–24.2%), 31.9% (15.3–46.8%), and 34.1 (12.8–52.4%), respectively. When looking at CVD mortality, we observed that eradicating hypertension would prevent up to 36.1% of deaths (18.0–51.8%) and acting against physical inactivity would reduce CVD mortality by 12.5% (1.00–23.7%). Finally, tobacco use cessation would result in a 44.1% (11.8–67.9%) reduction in respiratory disease mortality. Further reductions would be obtained by acting on underweight (41.5%; 11.8–67.9%%), physical inactivity (19.6%; 2.7–35.5) and alcohol consumption (33.4%; 6.7–5.7%).

**Table 2:** Population-attributable fractions for all-cause and cause-specific moratility attributable to modifiable risk factors

Exposure	Counts (%)	PAF (%) All-Cause	PAF (%) Cause-specific
HIV	400 (20.9%)	23.2% (17.4; 28.8)	47.9% (37.2–57.3) ^†^
Tobacco use	1261 (65.8%)	14.9% (3.6; 25.8)	44.1% (11.8; 67.9) ^¤^
Alcohol use	984 (51.4%)	18.1% (9.1; 26.8)	31.9% (15.3; 46.8) ^†^ 33.4% (6.7; 5.7) ^¤^
Physically Inactive	481 (25.0%)	9.7% (4.2; 15.1)	13.7% (3.0; 24.2) ^†^ 12.5% (1.00; 23.7) ^‡^ 19.6% (2.7; 35.5) ^¤^
Hypertension	907 (47.2%)	9.6% (1.3; 17.9)	36.1% (18.0; 51.8) ^‡^
Underweight	847 (44.1%)	17.5% (6.7; 27.9)	34.1% (12.8; 52.4) ^†^ 41.5% (11.8; 67.9) ^¤^

^†^ PAF due to infectious diseases; ^‡^ PAF due to CVD; ^¤^ PAF due to respiratory diseases

## Discussion

This study describes, for the first time, the mortality status and cause-of-death data of the North West Province, international PURE study site in South Africa. It furthermore describes the relative contribution of four main domains of health, namely locality, SES, lifestyle factors and prevalent disease, to all-cause and cause-specific mortality status. It also presents the population-attributable fractions of modifiable determinants in relation to population-level mortality risk. This study provides much-needed local empirical data necessary for the prioritisation and identification of appropriate health promotion, interventions and disease prevention programmes in South Africa.

Thirty per cent of the study participants had passed away during the 13-year follow-up, with a mean age of 59 years at death. The four most prevalent causes of death were infectious diseases (28.1%), followed by CVD (22.4%), respiratory diseases (11.6%) and cancer (11.1%). These results are broadly in line with the 2018 Global Burden of Disease (GBD) age-adjusted results (50–69 years age range) for South Africa (obtained from the IHME GHDx website) that report a 29.7% mortality rate due to infectious diseases (HIV/AIDS, tuberculosis, and other infectious diseases combined), and 18.8% due to CVD. According to the GBD, however, cancer-related mortality was higher at 17.1%, and respiratory disease was lower at 3.8%. Clear differences between national statistics and provincial and ethnic group-specific statistics have previously been reported for South Africa due to health inequalities between provinces and ethnic groups being at different stages of health transition [15]. The mean age at death due to infectious diseases (53 years) was, on average, seven years younger than the mean age at death due to respiratory and cancer-related deaths (60 years) and 12 years younger than deaths due to CVD (65 years).

Of the determinants investigated, the three main contributors to all-cause mortality were HIV infection, high SES and being underweight. This agrees with the fact that infectious diseases were also the most prevalent cause of death in this study sample, with HIV infection and underweight also being the main contributors to infectious disease mortality. The high prevalence of HIV infection is also reflected in national data, with both the Second Comparative Risk Assessment study for South Africa [8] and the 2019 Global Burden of Disease study [7], reporting unsafe sex to be the top risk factor for disability-adjusted life-years in all provinces of South Africa and the main risk factor to which HIV-related morbidity and mortality were attributed. Furthermore, individuals living with HIV frequently experience weight loss or have poor nutritional status resulting in protein-energy malnutrition. Research indicates that individuals living with HIV who are also underweight have a significantly higher all-cause mortality risk [16–19]. In 2005, few participants had access to antiretroviral therapy, as programmes to treat HIV were only initiated in 2005, and a large proportion of HIV-positive participants died early during the first ten years of follow-up [20]. Future risk assessment studies should hopefully reflect a more positive long-term effect of the national antiretroviral therapy roll-out by reducing the HIV-attributable burden.

There is general consensus in the literature that low SES is associated with increased mortality [21]. The reason why high SES was the second largest contributor to all-cause mortality in this study, may be explained by the definition for high SES applied. It may also be related to the fact that international literature mainly reflect data collected in high-income countries where NCDs are the main cause of death [22]. Also, studies define SES differently, e.g., some consider markers of both wealth and education such as occupation [21] and others education only [23], with further discrepancies regarding what level of education is defined as educated. Here high SES was defined as participants with high-school education above grade 9 and being employed, which would not be considered high SES in other countries. In this dataset, the association of high SES with all-cause mortality likely reflects an association between high SES and infectious disease mortality, as this is the most prevalent cause of death in this study and the only cause-specific mortality associated with increased SES. In support of this finding, the prevalence of high SES was much higher in the urban (75.2%) than the rural area (61.6%), which is in agreement with the fact that infectious diseases is the main cause of death in urban areas in low- and middle-income countries that are still in the earlier stage of the epidemiological transition [5].

The three main contributors to CVD mortality were hypertension, living in the urban environment and physical inactivity, with the two-fold increased CVD mortality risk of hypertension remaining in the mutually adjusted model. While hypertension is considered to be the leading cause of CVD and -related mortality worldwide, various studies have indicated that CVD mortality linked to hypertension was even more prevalent in individuals of African descent than in individuals of European descent [24–26]. This is thought to be related, in part, to impaired peripheral and cerebral micro- and macrovascular function both in terms of impaired vasodilatory capacity and exaggerated vasoconstrictor responsiveness [27] as well as salt sensitivity, volume-loading hypertension and the activity of the rennin-angiotensin system [28]. The increased CVD mortality risk related to urbanicity is not surprising, given that urbanisation significantly contributes to the increased NCD prevalence in low and middle-income countries. In these countries, urbanisation is typically associated with the development of urban slums, increased socio-economic disparity, poverty, obesity, hypertension, dietary changes (low fibre, high fat, and high energy content) and decreased physical activity [1, 3]. The beneficial relationship between physical activity and reduced CVD risk confirms data reported for the international PURE study [29] and other large prospective studies such as the United States National Health and Nutrition Examination Survey [30]. Sufficient physical activity exerts its protective effects through numerous biological mechanisms, such as improving cardiovascular function through enhanced oxygen delivery and simultaneously decreasing myocardial oxygen demand. Regular physical activity also improves glucose homeostasis and insulin sensitivity independent of body weight. In addition, recent research findings reported increased immune responses in persons performing regular physical activity [31, 32].

Our investigation is not limited to only identifying major determinants of all-cause and cause-specific mortality. Calculating the population-attributable fractions of modifiable determinants, we also showed where the focus of public health interventions should be placed. Specifically, we confirm that HIV eradication should be the main target of any public health intervention in the country. The complete eradication of HIV would avoid almost half of the deaths due to infectious disease and nearly a fourth of all-cause deaths. Not only HIV but also numerous other behavioural risk factors emerged as key to reducing the mortality burden in South Africa. Among these, we found that 10% of all-cause mortality is attributable to physical inactivity. Implementing supervised exercise strategies in at risk populations will increase the physical activity levels, address the control of blood pressure, improve the immune system in persons living with HIV and prevent the development of CVD in antiretroviral therapy users. Alcohol consumption and tobacco use, commonly acknowledged as major health problems in South Africa, contributed 15 and 18% [8], respectively, to the all-cause mortality burden. In addition, when looking at specific causes of mortality, we showed that these same risk factors were responsible for up to 30% of deaths. In particular, our results show that addressing hypertension should be the primary focus to reduce CVD deaths, as up to 36% of CVD deaths could be prevented by acting on hypertension alone. These strategies should focus both on treatment as well as improved early detection of hypertension. Respiratory mortality, on the other hand, can be reduced by 44.1% through tobacco use cessation. Currently the South African government is actively involved in numerous campaigns to reduce HIV and hypertension incidence as well as smoking cessation [13, 33]. However, our data indicates that the other above-mentioned behavioural determinants, alcohol consumption and physical inactivity, are equally important and should additionally be prioritised in health promotion and disease prevention interventions.

### Strengths and limitations

This study has numerous strengths. Firstly, we identified the main determinants of mortality and estimated their mortality burden in Black South Africans, an underrepresented population in epidemiological studies. Notably, our study is based on the South African arm of the PURE study, one of the continent’s most reliable data sets. The methodological strength of our work should also be highlighted, for we used outcomes based on validated and reliable national death records. From this perspective, we also highlight the use of advanced statistical tools. Finally, our results have significant practical application through showing where public health efforts should be focussed to produce the most profitable outcomes by means of mortality reduction. However, we also acknowledge the limitations of our work. First, there may be potential for error in the classification of T2DM since there is uncertainty regarding the use of HbA1c to diagnose T2DM in Africans [34]. Therefore, we decided to use HbA1C, fasting glucose and the use of oral hypoglycaemic agents to indicate diabetes. Second, physical activity by self-report is known for reporting bias, and future studies should collect objective physical activity data for more precise findings. Thirdly, we cannot exclude that the small number of deaths may have influenced our results by means of false negative outcomes. This most likely affected our investigation of cancer mortality, as none of the investigated risk factors demonstrated statistically significant associations with cancer mortality. Another potential limitation may be the lack of precision due to measurement errors, misreporting or even logistic and methodological problems affecting all the population-based research conducted on the African continent. Finally, being a well-phenotyped study, it allowed for the identification and control of many covariates; however, residual confounding cannot be excluded. In this regard, future large-scale studies based on a prospective design should be undertaken to better address the determinants of mortality and their public health impact on the African continent.

## Conclusion

An in-depth understanding of comparative risk assessment with regard to all-cause and cause-specific mortality provides evidence-based guidelines towards prioritising health promotion and disease prevention strategies. Here we demonstrate that in this Setswana-speaking community in the North West Province of South Africa, infectious diseases, and in particular HIV, was the leading cause of death, followed by CVD, respiratory and cancer mortality, confirming the presence of a double burden of disease (infection and NCD). Of the four domains of health, urban locality, high SES, prevalent disease (HIV and hypertension) and lifestyle factors (physical inactivity, tobacco use and alcohol consumption) all contributed in varying degrees to all-cause and cause-specific mortalities. While our data confirm the public health importance of addressing HIV and hypertension, it also highlights the importance of physical inactivity, tobacco use and alcohol consumption as focal points for public health strategies to produce the most efficient mortality reduction outcomes.

### Electronic supplementary material

Below is the link to the electronic supplementary material.

Supplementary Material 1

## Data Availability

The datasets used and/or analysed during the current study are available from the SA, North-West PURE study PI, Prof C Ricci (crisitan.ricci@nwu.ac.za), on reasonable request, following ethical approval by the NWU-HREC of the request.

## Abbreviations

95% CI: 95% confidence interval
AIDS: Acquired immunodeficiency syndrome
BMI: Body mass index
CVD: Cardiovascular disease
GBD: Global Burden of Disease
HbA1c: Glycated haemoglobin
HIV: Human immunodeficiency virus
HR: Hazard ratio
ICD10: International Classification of Diseases’ 10th revision
NCD: Non-communicable disease
PAF: population attributable fraction
PURE: Prospective Urban Rural Epidemiology study
SES: Socio-economic status
T2DM: Type 2 diabetes mellitus
