# Supplementary material for: Determinants of mortality status and population attributable risk fractions of the North West Province, South African site of the international PURE study
Source: Arch Public Health. 2024 Jul 5;82:102. doi: 10.1186/s13690-024-01336-y (PMC11225367; doi:10.1186/s13690-024-01336-y)
Supplement: Supplementary file 1 — Supplementary Material 1 [file 13690_2024_1336_MOESM1_ESM.docx]

Supplementary table 1. Hazard ratio for all cause and cause specific mortality performed by sex. The HR for BMI categories was performed considering normal weight (BMI = 18.5-25 kg/m2) as reference category.

| **Women** |  | **HR_1_ (95% CI)** |  | **HR_2_ (95% CI)** |  | **HR_3_ (95% CI)** |  | **HR_4_ (95% CI)** |  | **HR_5_ (95% CI)** |
| --- | --- | --- | --- | --- | --- | --- | --- | --- | --- | --- |
| Urban vs. Rural |  | 1.39 (1.10; 1.75) |  | 1.42 (0.92; 2.19) |  | 2.05 (1.23; 3.40) |  | 2.80 (1.24; 6.33) |  | 1.20 (0.58; 2.48) |
| High vs. low SES |  | 1.40 (1.09; 1.80) |  | 1.38 (0.85; 2.25) |  | 1.57 (0.94; 2.62) |  | 0.87 (0.42; 1.80) |  | 0.90 (0.43; 1.89) |
| Physically inactive |  | 1.49 (1.16; 1.91) |  | 1.72 (1.07; 2.76) |  | 2.05 (1.26; 3.33) |  | 1.66 (0.77; 3.59) |  | 1.02 (0.42; 2.43) |
| Ever smoke |  | 1.18 (0.93; 1.50) |  | 1.30 (0.82; 2.06) |  | 0.89 (0.55; 1.42) |  | 2.68 (1.09; 6.58) |  | 1.81 (0.80; 4.09) |
| Alcohol use |  | 1.49 (1.18; 1.88) |  | 1.77 (1.15; 2.72) |  | 1.21 (0.74; 1.96) |  | 2.95 (1.39; 6.24) |  | 1.74 (0.85; 3.59) |
| HIV |  | 3.15 (2.38; 4.18) |  | 9.53 (5.71; 15.9) |  | 1.25 (0.57; 2.76) |  | 1.96 (0.74; 5.21) |  | 2.02 (0.84; 4.86) |
| Hypertension |  | 1.08 (0.85; 1.37) |  | 1.25 (0.80; 1.96) |  | 1.67 (1.03; 2.85) |  | 0.91 (0.43; 1.91) |  | 0.57 (0.26; 1.24) |
| Type-II Diabetes |  | 0.94 (0.66; 1.34) |  | 0.30 (0.10; 0.97) |  | 0.97 (0.50; 1.91) |  | 0.22 (0.03; 1.61) |  | 0.52 (0.12; 2.20) |
| Underweight |  | 1.50 (1.07; 2.10) |  | 2.41 (1.27; 4.59) |  | 1.22 (0.58; 2.56) |  | 1.42 (0.56; 3.61) |  | 0.75 (0.28; 2.01) |
| Overweight |  | 0.89 (0.61; 1.29) |  | 0.89 (0.40; 1.94) |  | 1.15 (0.56; 2.36) |  | 0.63 (0.21; 1.87) |  | 0.86 (0.33; 2.22) |
| Obese |  | 0.76 (0.53; 1.08) |  | 0.72 (0.34; 1.53) |  | 0.91 (0.45; 1.85) |  | 0.31 (0.09; 1.04) |  | 0.33 (0.11; 1.01) |
| **Men** |  | **HR_1_ (95% CI)** |  | **HR_2_ (95% CI)** |  | **HR_3_ (95% CI)** |  | **HR_4_ (95% CI)** |  | **HR_5_ (95% CI)** |
| Urban vs. Rural |  | 1.27 (1.00; 1.60) |  | 1.09 (0.70; 1.70) |  | 1.49 (0.87; 2.55) |  | 1.12 (0.59; 2.14) |  | 1.65 (0.81; 3.33) |
| High vs. low SES |  | 1.62 (1.23; 2.14) |  | 1.71 (0.99; 2.95) |  | 1.20 (0.69; 2.10) |  | 0.97 (0.49; 1.94) |  | 2.60 (1.07; 6.33) |
| Physically inactive |  | 1.33 (1.03; 1.71) |  | 1.50 (0.93; 2.42) |  | 1.07 (0.60; 1.88) |  | 2.16 (1.10; 4.22) |  | 1.73 (0.86; 3.49) |
| Ever smoke |  | 1.40 (1.04; 1.89) |  | 1.50 (0.84; 2.67) |  | 1.54 (0.79; 2.99) |  | 1.79 (0.74; 4.31) |  | 0.99 (0.46; 2.13) |
| Alcohol use |  | 1.36 (1.02; 1.82) |  | 2.29 (1.21; 4.35) |  | 1.21 (0.66; 2.23) |  | 1.24 (0.58; 2.64) |  | 1.06 (0.49; 2.28) |
| HIV |  | 2.19 (1.65; 2.92) |  | 3.91 (2.44; 6.27) |  | 0.82 (0.32; 2.08) |  | 1.57 (0.66; 3.73) |  | 1.37 (0.52; 3.65) |
| Hypertension |  | 1.37 (1.08; 1.74) |  | 1.23 (0.79; 1.93) |  | 2.99 (1.63; 5.50) |  | 0.80 (0.41; 1.56) |  | 0.97 (0.49; 1.93) |
| Type-II Diabetes |  | 0.88 (0.53; 1.46) |  | 0.91 (0.33; 2.53) |  | 0.72 (0.22; 2.34) |  | 0.73 (0.17; 3.09) |  | 0.83 (0.20; 3.50) |
| Underweight |  | 1.49 (1.04; 2.13) |  | 1.88 (0.89; 3.95) |  | 0.57 (0.30; 1.06) |  | 9.62 (1.30; 71.0) |  | 3.43 (0.80; 14.7) |
| Overweight |  | 0.85 (0.51; 1.43) |  | 0.48 (0.13; 1.83) |  | 0.36 (0.13; 0.99) |  | 1.24 (0.08; 19.9) |  | 2.41 (0.44; 13.3) |
| Obese |  | 0.68 (0.29; 1.62) |  | Not estimable |  | 0.51 (0.12; 2.25) |  | Not estimable |  | 3.92 (0.55; 28.1) |
| **HR_1_**: Hazard ratio for all-cause mortality, **HR_2_**: Hazard ratio for infectious disease mortality, **HR_3_**: Hazard ratio cardiovascular disease mortality, **HR_4_**: Hazard ratio for respiratory disease mortality, **HR_5_**: Hazard ratio for cancer mortality | | | | | | | | | | |

Supplementary table 2. Hazard ratio for all cause and cause specific mortality performed after exclusion of participants who passed away during the first year of observation time (n = 1 878). The HR for BMI categories was performed considering normal weight (BMI = 18.5-25 kg/m2) as reference category.

|  |  | **HR_1_ (95% CI)** |  | **HR_2_ (95% CI)** |  | **HR_3_ (95% CI)** |  | **HR_4_ (95% CI)** |  | **HR_5_ (95% CI)** |
| --- | --- | --- | --- | --- | --- | --- | --- | --- | --- | --- |
| Urban vs. Rural |  | 1.35 (1.14; 1.61) |  | 1.30 (0.93; 1.80) |  | 1.70 (1.17; 2.47) |  | 1.81 (1.06; 3.09) |  | 1.43 (0.86; 2.39) |
| High vs. low SES |  | 1.37 (1.14; 1.66) |  | 1.36 (0.94; 1.97) |  | 1.32 (0.90; 1.93) |  | 0.79 (0.47; 1.33) |  | 1.42 (0.81; 2.47) |
| Physically inactive |  | 1.37 (1.13; 1.65) |  | 1.64 (1.15; 2.34) |  | 1.45 (0.99; 2.12) |  | 1.87 (1.10; 3.18) |  | 1.38 (0.80; 2.38) |
| Ever smoke |  | 1.37 (1.13; 1.67) |  | 1.54 (1.05; 2.26) |  | 1.20 (0.81; 1.78) |  | 2.44 (1.23; 4.86) |  | 1.28 (0.73; 2.26) |
| Alcohol use |  | 1.49 (1.23; 1.80) |  | 2.09 (1.44; 3.03) |  | 1.24 (0.84; 1.83) |  | 2.18 (1.20; 3.96) |  | 1.31 (0.75; 2.27) |
| HIV |  | 2.51 (2.03; 3.09) |  | 5.59 (3.93; 7.95) |  | 0.94 (0.49; 1.79) |  | 1.55 (0.75; 3.18) |  | 1.59 (0.81; 3.09) |
| Hypertension |  | 1.27 (1.06; 1.51) |  | 1.29 (0.92; 1.80) |  | 2.26 (1.50; 3.40) |  | 0.97 (0.58; 1.63) |  | 0.82 (0.49; 1.37) |
| Type-II Diabetes |  | 0.94 (0.70; 1.27) |  | 0.54 (0.25; 1.16) |  | 0.87 (0.47; 1.59) |  | 0.29 (0.07; 1.21) |  | 0.67 (0.24; 1.86) |
| Underweight |  | 1.46 (1.13; 1.88) |  | 2.02 (1.23; 3.31) |  | 0.75 (0.46; 1.22) |  | 2.81 (1.23; 6.39) |  | 1.30 (0.62; 2.70) |
| Overweight |  | 0.88 (0.64; 1.19) |  | 0.70 (0.36; 1.37) |  | 0.71 (0.41; 1.25) |  | 0.74 (0.25; 2.22) |  | 1.08 (0.46; 2.52) |
| Obese |  | 0.73 (0.53; 1.01) |  | 0.50 (0.25; 1.02) |  | 0.64 (0.36; 1.15) |  | 0.46 (0.13; 1.59) |  | 0.60 (0.22; 1.62) |
| **HR_1_**: Hazard ratio for all-cause mortality, **HR_2_**: Hazard ratio for infectious disease mortality, **HR_3_**: Hazard ratio cardiovascular disease mortality, **HR_4_**: Hazard ratio for respiratory disease mortality, **HR_5_**: Hazard ratio for cancer mortality | | | | | | | | | | |
